# Frequency of providing a palliative approach to care in family practice: a chart review and perceptions of healthcare practitioners in Canada

**DOI:** 10.1186/s12875-021-01400-4

**Published:** 2021-03-27

**Authors:** Erin Gallagher, Daniel Carter-Ramirez, Kaitlyn Boese, Samantha Winemaker, Amanda MacLennan, Nicolle Hansen, Abe Hafid, Michelle Howard

**Affiliations:** 1 Department of Family Medicine, McMaster University, David Braley Health Sciences Centre 100 Main Street West, 5th Floor, Hamilton, ON L8P 1H6 Canada; 2 Division of Palliative Care, McMaster University, David Braley Health Sciences Centre 100 Main Street West, 5th Floor, Hamilton, ON L8P 1H6 Canada; 3 Division of Palliative Care, Department of Medicine, University of Ottawa, 451 Smyth, Road Ottawa, Ottawa, ON K1H 8M5 Canada; 4 Department of Palliative Care, Bruyere Continuing Care, 43 Bruyère St, Ottawa, ON K1N 5C8 Canada

**Keywords:** Primary care, Palliative care, Inter-professional, Practice review

## Abstract

**Background:**

Most patients nearing the end of life can benefit from a palliative approach in primary care. We currently do not know how to measure a palliative approach in family practice. The objective of this study was to describe the provision of a palliative approach and evaluate clinicians’ perceptions of the results.

**Methods:**

We conducted a descriptive study of deceased patients in an interprofessional team family practice. We integrated conceptual models of a palliative approach to create a chart review tool to capture a palliative approach in the last year of life and assessed a global rating of whether a palliative approach was provided. Clinicians completed a questionnaire before learning the results and after, on perceptions of how often they believed a palliative approach was provided by the team.

**Results:**

Among 79 patients (mean age at death 73 years, 54% female) cancer and cardiac diseases were the top conditions responsible for death. One-quarter of patients were assessed as having received a palliative approach. 53% of decedents had a documented discussion about goals of care, 41% had nurse involvement, and 15.2% had a discussion about caregiver well-being. These indicators had the greatest discrimination between a palliative approach or not. Agreement that elements of a palliative approach were provided decreased significantly on the clinician questionnaire from before to after viewing the results.

**Conclusions:**

This study identified measurable indicators of a palliative approach in family practice, that can be used as the basis for quality improvement.

**Supplementary Information:**

The online version contains supplementary material available at 10.1186/s12875-021-01400-4.

## Background

There is increasing evidence revealing the benefits of palliative care for patients with chronic, progressive life-limiting illness [1–6]. Fewer people are dying suddenly and most will live for a period of time with high healthcare and other needs [7]. Despite this knowledge, efforts to integrate, describe and evaluate palliative care in and across the healthcare system have not achieved the level of uptake and sustainability required [8–12]. Indeed, the World Health Organization has called for standardized access to palliative care as a human right [13]. Countries including Canada and the U.S. have taken steps to create strategies to help organizational change and planning to support policy and program decision making [14, 15]. Aging Canadians experience access and equity challenges, with only 15% receiving home palliative care and most palliative care being initiated due to unplanned hospital admissions or emergency department visits [16]. This promotes a sense of urgency for solutions [17].

Although government prioritization of palliative care is a promising development and it is recognized that primary care can contribute to addressing the majority of palliative care needs in the population [18], there remains a lack of conceptual clarity around certain aspects, including the differentiation of primary and specialist levels of palliative care, what they are comprised of and when each is needed [19, 20]. The phrase *palliative approach* has gained traction due to connotations of activities or tasks (not just philosophy) applicable to all care professionals (not just palliative care specialists) in a manner consistent with the philosophy of palliative care, regardless of whether care is labelled or approached as such [19, 21]. Sawatzky and colleagues identified three overarching themes delineating a *palliative approach*: (1) upstream orientation towards the needs of people who have life-limiting conditions and their families, (2) adaptation of palliative care knowledge and expertise, (3) operationalization of a *palliative approach* through integration into systems and models of care that do not specialize in palliative care [9].

In Canada and elsewhere, it is recognized that family medicine is a specialty in which a primary palliative approach would be ideally situated [22, 23] due to the provision of comprehensive care across the lifespan [24]. Investment in primary care health service delivery improves the overall health of populations and reduces inequity in healthcare distribution and health outcomes within populations [25]. The principles and activities of family medicine contribute to outcomes for patients with progressive life-limiting illness through coordination and continuity of care, care decision-making, care planning and referral to specialist care as needed [26–29]. Continuity of care with primary care providers has been shown to be valued by patients with chronic, progressive life-limiting illness [26, 30–32] and is cost-effective for the system [33, 34].

If healthcare systems are to respond to population needs for palliative care by promoting a primary level palliative approach, this care must be both conceptually clear and operationally defined and measurable. Measuring and supporting high quality primary care remains a challenge [25]. Tools and competencies exist to support family medicine in adopting a primary level palliative approach [11, 23, 35]. Nevertheless, there is evidence that generalists are not certain how to incorporate the approaches into practice [36]. Attempts to codify and measure whether and how elements of a palliative approach are applied in this setting have been limited [21, 28, 31].

To address the gap between conceptual descriptions and competencies, and observable measurable care processes in practice, in our previous work, we qualitatively described a *palliative approach to care* (PAC) in primary care, from the perspective of primary care physicians and nurses caring for patients with progressive life-limiting illnesses [2]. In this study, we sought to measure the extent to which PAC is provided in a large, urban inter-professional family health team (FHT) setting in one city in Canada. To assess the extent to which the outcomes resonated as a potential quality improvement opportunity, we also assessed the clinicians’ perception of how consistently the FHT provides elements of PAC, before and after learning about the results.

## Methods

We conducted a cross-sectional study to determine, by chart review, how consistently one FHT was implementing elements of PAC with patients in their last year of life. We chose the last year of life to align with literature showing sharp increases in levels of disability in the last year of life [37], the frequent reference to the last year of life in recommendations to initiate palliative care [38], and recognizing that a palliative approach to care should be incorporated from the time of diagnosis of a progressive life-limiting illness [12].

The study was conducted in an FHT made up of two multi-physician family medicine clinics in one city, serving approximately 40,000 patients. There are 40 family physician practices, diverse allied health professionals who work between practices and 80 family medicine residents. One hundred patient charts were randomly selected among 192 patients who died from January 1^st^ to December 31^st^, 2017. With inter-rater reliability being previously established, charts were equally divided between 2 reviewers for data extraction. Inclusion criteria were patients who had been a member of the practice for at least 1 year prior to their death and had a chronic, progressive life-limiting illness. Exclusion criteria included: orphan patients (admitted to the practice at end of life for the sole purpose of palliative care and not having a previously identified family doctor); sudden and unexpected deaths unrelated to a chronic progressive life-limiting illness; and patients residing in a long-term care facility for over 6 months of the last year of their life.

We created a chart review tool to capture the elements of PAC in primary care (Additional file 1). To develop the tool, we combined the three aforementioned conceptual domains of a palliative approach, and three core elements of published definitions of what constitutes palliative care from another systematic review. The three conceptual domains (early upstream approach, adaptation of palliative care knowledge and expertise to other settings, integration into systems) [19] and three elements of the definition (whole person care, quality of life focus, mortality acknowledgement) [9] were linked to the operational items/elements in the chart review form to create a tool for evaluating PAC in primary care. Categories in the final chart audit form included advance care planning topics documented, symptoms documented, type of clinicians involved in care, frequency of telephone contacts and home visits, involvement of formal home care services and attention to caregiver involvement and well-being. For descriptive purposes we also collected information on demographics, health conditions and illnesses most responsible for death.

Since there is no current operational definition of a palliative approach, the chart reviewers provided an overall assessment of their judgement of whether the care given by the family practice in the last year of life aligned with PAC. To make this assessment of having received PAC, patients required clinical documentation of elements mapping to all 3 domains. For practicality and to ensure the focus was on the efforts of the primary care team, documentation considered included the chart’s running commentary and the cumulative patient profile only. Consultant notes, messages and external documents were not considered because we wanted to capture care provided by the family practice, and because it was not practicable to review the volume of information. The timeframe for the chart review and assessment of a palliative approach was one year prior to the patient’s death.

For reasons of feasibility, we randomly selected 100 charts to review. Patient characteristics and outcomes of interest were described using mean (standard deviation) for continuous variables and count (percent) for categorical variables. For comparisons of outcomes between patients who did and did not receive a PAC, independent sample t-tests (for continuous data) and Pearson’s Chi-squared tests (for categorical data) or Fisher’s exact tests (for categorical data with expected cell sizes < 5) were used. Haldane’s correction was applied to contingency tables with observed zero values. Statistical significance of comparisons was indicated by a *p*-value < 0.05 (two-tailed) for the test. For comparisons of proportions, an absolute difference of 30% between groups could be detected with 80% power, and there was greater than 80% power to detect a mean difference of 1.0 between groups for continuous variables.

Following data analyses, results were presented to clinicians within the FHT (*N* = 58) in a group meeting. A pre- and post- questionnaire was administered immediately before and then after the presentation, to determine perceptions around how consistently clinicians perceived they delivered the elements of a palliative approach. The questionnaire was developed by this study and has not been published elsewhere (Additional file 2). The survey included 8 questions and used 7-point Likert scale responses (1 = infrequently, 7 = regularly). The difference in mean scores for each question were compared by the paired t-test. Analyses were conducted using SAS© software, version 9.4 for Windows.

The STROBE checklist for cross-sectional studies was used. Ethics approval for the chart review was received by the Hamilton Integrated Research Ethics Board for this project (File number 2017–4204-C). We did not seek ethics approval for the anonymous staff questionnaire as this was undertaken to inform potential quality improvement efforts locally, as per Canada’s Tri-Council Policy Statement: Ethical Conduct for Research Involving Humans -TCPS 2 (2018) Article 2.5. We explained the purpose of the questionnaire and informed staff that completion was voluntary. Individual respondent consent was not elicited and was assumed by completion of the questionnaire.

## Results

Of the 100 randomly selected patients, 13 were excluded because they were patients without a family physician only transferred to the practice near end of life, four died suddenly of reasons unrelated to chronic life-limiting illness, and four were transferred to a long-term care institution. The remaining seventy-nine patients met inclusion criteria for the chart review. The mean age at death was 73.1 years; 54.4% were female; 79.7% had an unpaid caregiver recognized in the chart. The most common life-limiting diagnoses were cancer (49.4%) and end-stage cardiac diseases (40.5%). Over half of patients had a non-cancer diagnosis as the most attributable cause of death (Table 1).

**Table 1:** Characteristics of the 79 decedents from the Family Health Team

**Characteristic**	**N (%) or mean, standard deviation (SD)**
Mean (SD) age at death, y	73.1 (14.7)
Age range, y	37 to 99
Female sex	43 (54.4)
Marital status	
Not in a relationship	18 (22.8)
In a relationship (not living together)	3 (3.8)
Married or common-law (living together)	35 (44.3)
Not documented in chart	23 (29.1)
Education status	
Did not complete high school	1 (1.3)
Completed high school	0 (0.0)
Had some university education or completed a community college, technical college, or post-secondary program (ex. trade, technical, or vocational school)	2 (2.5)
University degree (ex. BA, BSc, BSN)	5 (6.3)
Graduate degree (ex. MD, DDS, DMD, DVM, OD, Master’s, or PhD)	2 (2.5)
Not documented in chart	69 (87.3)
Residence status	
At home (apartment/townhouse/bungalow)	60 (75.9)
Retirement residence	4 (5.1)
Supportive housing (group home/assisted living)	2 (2.5)
Long-term care or nursing home	1 (1.3)
Vulnerable housing (shelter/transition home/no home/subsidized housing)	0 (0.0)
Not documented in chart	12 (15.2)
Living alone prior to death	
Yes	16 (20.3)
No	43 (54.4)
Not documented in chart	20 (25.3)
Living with their caregiver	
Yes	42 (53.2)
No	13 (16.5)
Not documented in chart	24 (30.4)
Identified a caregiver in their chart	
Yes	63 (79.7)
No	16 (20.3)
Documented caregiver presence at office visits	
Yes	47 (59.5)
No	32 (40.5)
Documented caregiver’s concerns being addressed	
Yes	39 (49.4)
No	40 (50.6)
Documented caregiver’s well being addressed	
Yes	12 (15.2)
No	67 (84.8)
Documentation of chronic life-limiting illness	
Cancer	39 (49.4)
Cardiac disease	32 (40.5)
Mental health	18 (22.8)
Lung disease	12 (15.2)
Frailty	11 (13.9)
Renal disease	10 (12.7)
Dementia	9 (11.4)
Liver disease	7 (8.9)
Stroke	6 (7.6)
Neurodegenerative disease	1 (1.3)
Other	7 (8.9)
Illness most responsible for death	
Cancer	33 (41.8)
Cardiac disease	16 (20.3)
Mental health	3 (3.8)
Stroke	3 (3.8)
Frailty	2 (2.5)
Liver disease	2 (2.5)
Lung disease	2 (2.5)
Dementia	1 (1.3)
Neurodegenerative disease	1 (1.3)
Renal Disease	1 (1.3)
Other	15 (19.0)
Documented chronic life-limiting illnesses, mean (SD)	1.9 (0.9)

There was variability across care processes, for example, from 1.3% (evidence of involvement of an FHT social worker in care) to 44.3% (evidence of involvement of a FHT nurse). The care processes documented for most patients included addressing physical symptoms (89.9% of patients), followed by documentation of caregivers being present at visits (59.5%) and caregiver concerns documented (49.4%) (Table 2). Infrequent care processes included use of symptom assessment tools (6.3%) and discussion of cultural or religious concerns (e.g., rituals or beliefs that would impact care) regarding end of life (1.3%).

**Table 2:** Processes of care documented in decedents (*n* = 79) who did versus did not receive a palliative approach to care

**Variables documented in electronic medical record**	Overall (*n* = 79), n (%)	**Did the patient receive a palliative approach to care?**		*P*-value^ab^
		Yes (*N* = 20), n (%)	No (*N* = 59), n (%)	
**Mortality Acknowledgement**				
Advance Care Planning topics addressed and documented:				
Goals of care for treatment decisions (to pursue a treatment or not and why)	42 (53.2)	18 (90)	24 (40.7)	< .01
Understanding of severity of illness (illness awareness)	32 (40.5)	17 (85)	15 (25.4)	< .01
Values/beliefs/priorities moving forward	26 (32.9)	18 (90)	8 (13.6)	< .01
Do-Not-Resuscitate & Do-Not-Resuscitate Confirmation Form	20 (25.3)	15 (75)	5 (8.5)	< .01
Power of Attorney for Personal Care & Substitute Decision Makers	18 (22.8)	9 (45)	9 (15.3)	.01
Desired place of death	13 (16.5)	11 (55)	2 (3.4)	< .01
Prognosis	11 (13.9)	11 (55)	0 (0)	< .01
Will	2 (2.5)	2 (10)	0 (0)	.06
Funeral arrangements	2 (2.5)	2 (10)	0 (0)	.06
Other	5 (6.3)	2 (10)	3 (5.1)	.60
**Quality of Life**				
Homecare involvement documented:				
Nurse	32 (40.5)	17 (85)	15 (25.4)	< .01
Personal Support Worker	22 (27.8)	10 (50)	12 (20.3)	.01
Occupational Therapist	8 (10.1)	5 (25)	3 (5.1)	.02
Physiotherapist	4 (5.1)	2 (10)	2 (3.4)	.26
Psychosocial/Spiritual Advisor	3 (3.8)	3 (15)	0 (0)	.01
Registered Dietitian	2 (2.5)	1 (5)	1 (1.17)	.38
Other	12 (15.2)	1 (5)	11 (18.6)	.28
Quality of Life focused symptom discussions documented:				
Physical Symptoms	71 (89.9)	19 (95)	52 (88.1)	.67
Delirium	8 (10.1)	8 (40)	0 (0)	< .01
Symptom Assessment Tools used	5 (6.3)	2 (10)	3 (5.1)	.60
Other	1 (1.3)	1 (5)	0 (0)	.25
**Whole-person Care**				
Primary care involvement documented:				
Most Responsible Physician	68 (86.1)	20 (100)	48 (81.4)	.06
Medical Resident	59 (74.7)	18 (90)	41 (69.5)	.08
Nurse	35 (44.3)	14 (70)	21 (35.6)	.007
Pharmacist	17 (21.5)	4 (20)	13 (22.0)	1.00
Occupational Therapist	10 (12.7)	1 (5)	9 (15.3)	.44
Registered Dietitian	5 (6.3)	0 (0)	5 (8.5)	.32
Physiotherapist	2 (2.5)	0 (0)	2 (3.4)	1.00
Social Worker	1 (1.3)	1 (5)	0 (0)	.25
Other	23 (29.1)	5 (25)	18 (30.5)	.64
Caregiver involvement and status documented:				
Caregiver presence documented	47 (59.5)	18 (90.0)	29 (49.2)	.01
Caregiver concerns documented	39 (49.4)	18 (90.0)	21 (35.6)	< .01
Caregiver well-being documented	12 (15.2)	9 (45.0)	3 (5.1)	< .01
Whole-person care focused symptom discussions documented:				
Existential/psychosocial/spiritual concerns	33 (41.8)	16 (80)	17 (28.8)	< .01
Safety concerns	26 (32.9)	11 (55)	15 (25.4)	.02
Financial concerns	17 (21.5)	7 (35)	10 (16.9)	.11
Cultural concerns	1 (1.3)	0 (0)	1 (1.7)	1.00
Phone care documented as ‘Often’ (5 or more phone calls)^d^	33 (43.4)	15 (75)	18 (32.1)	< .01
Documented number of home visits in final year of life, mean (SD)	2.3 (7.1)	4.0 (5.1)	1.7 (7.6)	.21^c^

^a^Pearson’s Chi-Squared Test

^b^Fisher’s Exact Test for categorical data with expected cell sizes < 5

^c^Independent samples t-test

^d^Overall sample of *N* = 76 due to incomplete chart reviews

Twenty (25%) of the patients were assessed as having received a PAC by our criteria. PAC patients tended to be older compared to non-PAC patients (mean age 78.3 vs 71.4 years; *p*=0.07); more likely to be female (60% vs 52.5%; *p*=0.69) and have a higher number of documented life-limiting illnesses (mean 2.2 versus 1.8; *p*=0.18), although these differences were not statistically significant.

Patients with cancer (60.0% versus 45.8%; *p* = 0.27), cardiac disease (50.0% versus 37.3%; *p* = 0.32) or renal disease (25.0% versus 8.5%; *p* = 0.11) were more likely to receive PAC than those without the conditions, although the differences were not statistically significant.

The care processes with the greatest difference between PAC and non-PAC patients were: discussion about values, beliefs, and priorities as part of advance care planning (90% vs 13.6%; *p* < 0.01); community home care nurse involvement (85% vs 25.4%; *p* < 0.01); FHT practice nurse involvement (70% vs 35.6%; *p* < 0.01); documentation of existential, psychosocial or spiritual concerns (80% vs 28.8%; *p* < 0.01); and documentation related to addressing caregiver well-being (45.0% vs 5.1%; *p* < 0.01). PAC patients were also more likely than non-PAC patients to receive frequent (5 or more) phone calls (75% versus 32.1%; *p* < 0.01). Compared to patients who did not receive PAC, PAC patients tended to have more home visits (mean 4 versus 1.7 visits; *p* = 0.21). Allied health professionals of the FHT were infrequently involved.

Among the FHT clinicians who attended the presentation of results, 33.3%% were physicians, 29.2% were residents, 16.7% were nurses, 14.6% were allied health professionals and 6.3% were support staff. After the presentation, the mean ratings of perceived consistency of delivering a palliative approach decreased statistically significantly (*p* < 0.05) for all questions (Table 3). The care process rated by clinicians as lowest before seeing results was having goals of care conversations (mean = 4.3, SD = 1.2). The item with the largest reduction in perceived consistency was for involvement of FHT allied health professionals (mean before = 5.1, SD = 1.5; mean after = 3.5 SD = 1.6).

**Table 3:** Rating of consistency of providing a palliative approach (1 = infrequently, 7 = regularly) by Family Health Team

**Survey question**	**Mean, standard deviation**		***P*-value**
	**Before**	**After**	
How consistently does your clinic provide a palliative approach to care for patients with chronic, progressive, life-limiting illnesses?	5.0 (1.2)	3.8 (1.3)	< .01
How consistently does your clinic provide whole-person care to patients with chronic, progressive, life-limiting illnesses?	5.5 (1.1)	4.4 (1.5)	< .01
How consistently does your clinic provide a quality-of-life focus to patients with chronic, progressive, life-limiting illnesses?	5.5 (1.1)	4.3 (1.5)	< .01
How consistently does your clinic provide mortality acknowledgment (including preparation for death, advance care planning and goals of care discussions) to patients with chronic, progressive, life-limiting illnesses?	4.3 (1.2)	3.3 (1.5)	< .01
How consistently does your clinic use the allied health team (from your clinic) for patients with chronic, progressive, life-limiting illnesses?	5.1 (1.5)	3.5 (1.6)	< .01
How consistently does your clinic use external allied clinician resources (from the community) for patients with chronic, progressive, life-limiting illnesses?	4.7 (1.4)	4.2 (1.5)	0.01
How consistently does your clinic address the needs of the caregiver for patients with chronic, progressive, life-limiting illnesses?	4.7 (1.4)	3.9 (1.4)	< .01
How consistently does your clinic assess and manage symptoms of patients with chronic, progressive, life-limiting illnesses?	5.5 (1.1)	5.0 (1.2)	.01

## Discussion

In this study of the care of patients in their last year of life by their family practice, one-quarter of patients were assessed as having received PAC. PAC was evident for patients with cancer and non-cancer illnesses. Documentation of advance care planning discussions, clinic and community homecare nursing involvement, addressing psychosocial and spiritual needs, and addressing caregiver wellness were care processes that distinguished between patients receiving PAC and not receiving PAC. In previous research, family physicians reported feeling capable of meeting the palliative needs of many patients [2, 39], however they have also reported doing so without a specific plan or approach [40]. Patients with progressive life-limiting illness have varying needs over the last year of life, beyond medical treatment for physical symptoms. The presence of additional aspects of care including discussions about goals of care, mobilizing more resources to care for the patient in the community and involving and supporting caregivers were also present for patients receiving PAC.

It was interesting that symptoms were addressed for a high proportion of patients as indicated in notes in the chart, however specific assessment tools were rarely used. This could be due to the lack of routine use of a specific set of palliative care tools in the common electronic medical record used by the practices. Routine use of a structured tool may trigger a broader assessment of symptoms including psychosocial and spiritual issues [41], however in primary care their use is generally low [42] and this represents a potential area for improvement.

Historically the evidence for palliative care has emerged from studies of patients with cancer, however there is increasing recognition of the palliative needs of people with non-cancer diagnoses [6]. Our study reveals the application of a palliative approach to care for patients with several other chronic, progressive life-limiting illnesses. Patients with cancer tend to have a defined and short terminal phase, characterized by step-wise drops in function and they are more likely to receive formal palliative care services and for longer periods before death, compared to non-cancer illnesses [43, 44]. The duration of non-cancer palliative needs can be longer and less certain than that for cancer [37, 44] and the needs of patients with non-cancer life-limiting illnesses can be less intense over this prolonged period, making early identification a challenge. This could be one reason that only 25% of patients received a PAC in their last year of life, as most deaths were non-cancer related.

Family medicine, being a community-based discipline that prioritizes the patient-physician relationship, is in a unique position to build upon the care processes that we have operationalized for a PAC [45]. Presentation of the study results shifted the FHT clinicians’ perception of the consistency with which PAC is applied by the clinic and the new information provided specific and tangible areas to focus on for improvement. The fact that FHT clinicians changed their perceptions suggests that although the care is often present, there is opportunity to be more intentional about naming and providing it.

This study has some limitations. The study was conducted in one setting and may not be representative of all Canadian family practices, however the clinics are part of a large practice-based research network with approximately 40 physicians. Our consensus process to define the elements of a palliative approach in family practice through chart review was done with experts from a single academic institution rather than multiple institutions and jurisdictions. However, the team has specialized knowledge through clinical practice, education, and health system leadership in the area of integration of a primary palliative approach in family practice. We were not able to assess if care was proactive and responsive to patient and caregiver needs, therefore we cannot comment on quality of care. Finally, the judgement of whether PAC was present reflected an overall expert assessment rather than an empirically driven approach, and it was not independent of domains in the chart review. It will be important to empirically validate the care processes that comprise a palliative approach in a larger multi-centre study.

## Conclusions

Our study operationalized care indicators that can be used to describe the extent to which PAC is applied, which may help efforts to ensure equitable access and quality care. Measuring what family physicians and other primary care clinicians are already doing as part of routine practice highlights processes that can be leveraged and enhanced. The distinguishing processes of PAC we observed reinforced conceptual definitions of PAC and are within the scope of family medicine and did not require specialized palliative medicine physicians or additional resources that are not already available in family practice. Our results, which highlight how primary care can provide a palliative approach as part of routine practice, can benefit models of health care that seek to optimize the relationship between primary care and specialized palliative care resources and community resources. The findings can also assist these groups to implement an integrated palliative approach to care across different sectors. Future research will need to explore patient, family member and caregiver perceptions around the operationalized elements of a palliative approach to care and how they are experienced. This could shape any future interventions and identified outcomes measures.

## Supplementary Information

**Additional file 1.** Chart Review Tool. This chart review tool was developed by this study to capture the elements of PAC in primary care.

**Additional file 2.** Pre- & Post-Chart Review Questionnaire. The questionnaire was developed by this study and has not been published elsewhere. 

## Data Availability

The anonymized data used and analysed during the current study are available from the corresponding author on reasonable request.

## Abbreviations

FHT: Family Health Team
PAC: Palliative approach to care
