# Supplementary material for: Frequency of providing a palliative approach to care in family practice: a chart review and perceptions of healthcare practitioners in Canada
Source: BMC Fam Pract. 2021 Mar 27;22:58. doi: 10.1186/s12875-021-01400-4 (PMC8005234; doi:10.1186/s12875-021-01400-4)
Supplement: Supplementary file 1 — Additional file 1. Chart Review Tool. This chart review tool was developed by this study to capture the elements of PAC in primary care. [file 12875_2021_1400_MOESM1_ESM.pdf]

CHART ABSTRACTION FORM

Range of date being reviewed (date of death minus 1 year):_________________________

A. Demographic Variables:

1. Age at death: ______________ years

2. Sex:

- ❑ Female
- ❑ Male

3. Education:

- ❑ Did not complete secondary school or high school
- ❑ Completed secondary or high school
- ❑ Had some university education or completed a community college, technical college, or post-secondary program (for example; trade, technical or vocational school)
- ❑ University degree (for example; BA, BSc, BSN)
- ❑ Graduate degree (for example; MD, DDS, DMD, DVM, OD, Master's, or PhD)
- ❑ *Specify*: ____
- ❑ Information not located in chart

4. Relationship Status at the time of death:

- ❑ Married or living as married
- ❑ Widowed
- ❑ Never married
- ❑ Divorced or separated; not remarried
- ❑ *Specify*: ____

5. Living Arrangement prior to transfer to place of death (if applicable):

- ❑ At home (apartment, townhouse, bungalow, etc)
- ❑ Retirement Residence
- ❑ Long-Term Care or Nursing Home
- ❑ Vulnerable Housing (eg. Shelter, transition home, no home)
- ❑ Other (e.g *specify*):
  ____

6. Primary Caregivers (e.g. informal care team, family):

Name	Relationship

7. Chronic Life-Limiting Illnesses contributing to death: *check all that apply*

- ❑ Cancer
- ❑ Cardiac disease (CAD, CHF, etc.)
- ❑ Stroke
- ❑ Lung disease (COPD, ILD, etc.)
- ❑ Dementia
- ❑ Neurodegenerative disease (ALS, MS, Multisystem Atrophy, Parkinson, HD, etc)
- ❑ Liver disease
- ❑ Renal disease
- ❑ Frailty
- ❑ Other: ____
- ❑ Other: ____

8. Chronic Life-Limiting Illness Most Responsible For Death:

- ❑ Cancer
- ❑ Cardiac disease (CAD, CHF, etc.)
- ❑ Stroke
- ❑ Lung disease (COPD, ILD, etc.)
- ❑ Dementia
- ❑ Neurodegenerative disease (ALS, MS, Multisystem Atrophy, Parkinson, HD, etc)
- ❑ Liver disease
- ❑ Renal disease
- ❑ Frailty
- ❑ Other: ____

B. Billing

**Code Used**	**Dates when code was used**
Case management<br>❑ G512	
Home Care Application<br>❑ K070	
Death Certificate, following patient to end<br>❑ A771	
Home visit codes<br>❑ B966<br>❑ B998<br>❑ A901	
Palliative Care Support >20 mins<br>❑ K023	
Counselling of relatives<br>❑ K015	
❑ G511	
Consult Codes<br>❑ K730<br>❑ K731<br>❑ K738<br>❑ K739	

C. Advance Care Planning

Take into consideration only if and when these conversations occurred. Include date for all relevant variables.

Variable	Date First Occurred (DD/MM/YYYY)	Repeat Conversation
❑ Understanding of severity of illness (illness awareness)		❑ yes
❑ Prognosis		❑ yes
❑ Values/beliefs/priorities moving forward (ACP)		❑ yes
❑ Goals of care for treatment decisions (to pursue a treatment or not and why)		❑ yes
❑ Desired place of death		❑ yes
❑ DNR/DNR-C		❑ yes
❑ Will		❑ yes
❑ POA/SDM		❑ yes
❑ Funeral arrangements		❑ yes

D. Home Care

(Home visits by practitioners)

Type of service	Date Service Started (DD/MM/YYYY)

E. Community Services/Resources

(CCAC or other community referrals, any evidence that it happened, date, f/u with patients))

Type of service	Date (DD/MMM/YYYY)

F. Caregiver/Informal Supports

(Caregiver presence in encounters/ caregiver issues/ well being addressed)

Variable	Occurred?
Present during >1 encounters with patient	YES<br><br>NO
Caregiver concerns/well being discussed	YES<br><br>NO
Caregiver concerns/well being readdressed	YES<br><br>NO

G. Symptoms

Type of service	Provider	Date (DD/MMM/YYYY)
❑ Were physical symptoms discussed?		
❑ Were they reassessed?		
❑ Were medications prescribed?		
❑ Were any tools used to assess symptoms?<br>*Specify:*________________________________<br>*Frequency*:______________________________		
❑ Existential/ Spiritual Concerns		

❑ Cultural Concerns		
❑ Financial Concerns		
❑ Other psychosocial concerns		

H. Continuity of Care

Variable	No. of encounters	Type of Encounter	
MRP encounters		❑ Phone<br>Date(s):	❑ In-Person<br>Date(s):
Resident encounters		❑ Phone<br>Date(s):	❑ In-Person<br>Date(s):
Nurse practitioner encounters		❑ Phone<br>Date(s):	❑ In-Person<br>Date(s):
OT encounters		❑ Phone<br>Date(s):	❑ In-Person<br>Date(s):
PT encounters		❑ Phone<br>Date(s):	❑ In-Person<br>Date(s):
Pharmacy encounters		❑ Phone<br>Date(s):	❑ In-Person<br>Date(s):
Social work encounters		❑ Phone<br>Date(s):	❑ In-Person<br>Date(s):

Nurse encounters		❑ Phone<br>Date(s):	❑ In-Person<br>Date(s):
Palliative care consultant encounters		❑ Phone<br>Date(s):	❑ In-Person<br>Date(s):
Home visits		Date:	
Clinic visits		Date:	
Hospital visits		Date:	
Hospice visits		Date:	
LTC visits		Date:	

I. Specialist Care

**Type of Specialist**	**Name**	**Date (DD/MMM/YYYY)**

J. Other

Variable
Place of death
Length of stay at place of death
On call encounters
ER encounters
Cause of death
Other Comments:
