# Supplementary material for: Frequency of providing a palliative approach to care in family practice: a chart review and perceptions of healthcare practitioners in Canada
Source: BMC Fam Pract. 2021 Mar 27;22:58. doi: 10.1186/s12875-021-01400-4 (PMC8005234; doi:10.1186/s12875-021-01400-4)
Supplement: Supplementary file 2 — Additional file 2. Pre- & Post-Chart Review Questionnaire. The questionnaire was developed by this study and has not been published elsewhere. [file 12875_2021_1400_MOESM2_ESM.docx]

**Additional File 1: Questionnaire**

**PRE - Presentation Questionnaire ID#_______**

A palliative approach to care can be described as the following: “Non-specialists adapt palliative care knowledge and expertise, integrate this knowledge into other systems and models of care, and apply it upstream in the care of patients with life-limiting illnesses.” – Sawatzky et al, 2016

Based on the above definition, how consistently does your clinic provide a **palliative approach to care** for patients with chronic, progressive, life-limiting illnesses (circle one)?

| **1** | **2** | **3** | **4** | **5** | **6** | **7** |
| --- | --- | --- | --- | --- | --- | --- |
| Infrequently |  |  | Occasionally |  |  | Regularly |

How consistently does your clinic provide **whole-person care** to patients with chronic, progressive, life-limiting illnesses?

| **1** | **2** | **3** | **4** | **5** | **6** | **7** |
| --- | --- | --- | --- | --- | --- | --- |
| Infrequently |  |  | Occasionally |  |  | Regularly |

How consistently does your clinic provide a **quality-of-life focus** to patients with chronic, progressive, life-limiting illnesses?

| **1** | **2** | **3** | **4** | **5** | **6** | **7** |
| --- | --- | --- | --- | --- | --- | --- |
| Infrequently |  |  | Occasionally |  |  | Regularly |

How consistently does your clinic provide **mortality acknowledgment** (including preparation for death, ACP and GOC discussions) to patients with chronic, progressive, life-limiting illnesses?

| **1** | **2** | **3** | **4** | **5** | **6** | **7** |
| --- | --- | --- | --- | --- | --- | --- |
| Infrequently |  |  | Occasionally |  |  | Regularly |

How consistently does your clinic use the **allied health team** (from your clinic) for patients with chronic, progressive, life-limiting illnesses?

| **1** | **2** | **3** | **4** | **5** | **6** | **7** |
| --- | --- | --- | --- | --- | --- | --- |
| Infrequently |  |  | Occasionally |  |  | Regularly |

How consistently does your clinic use **external allied clinician resources** (from the community) for patients with chronic, progressive, life-limiting illnesses?

| **1** | **2** | **3** | **4** | **5** | **6** | **7** |
| --- | --- | --- | --- | --- | --- | --- |
| Infrequently |  |  | Occasionally |  |  | Regularly |

How consistently does your clinic address the **needs of the caregiver** for patients with chronic, progressive, life-limiting illnesses?

| **1** | **2** | **3** | **4** | **5** | **6** | **7** |
| --- | --- | --- | --- | --- | --- | --- |
| Infrequently |  |  | Occasionally |  |  | Regularly |

How consistently does your clinic **assess and manage symptoms** of patients with chronic, progressive, life-limiting illnesses?

| **1** | **2** | **3** | **4** | **5** | **6** | **7** |
| --- | --- | --- | --- | --- | --- | --- |
| Infrequently |  |  | Occasionally |  |  | Regularly |

Please indicate (check) your role in the clinic:

- Physician
- Resident
- Nurse
- Allied Health
- Support Staff

**POST - Presentation Questionnaire ID#_______**

A palliative approach to care can be described as the following: “Non-specialists adapt palliative care knowledge and expertise, integrate this knowledge into other systems and models of care, and apply it upstream in the care of patients with life-limiting illnesses.” – Sawatzky et al, 2016

Based on the above definition, how consistently does your clinic provide a **palliative approach to care** for patients with chronic, progressive, life-limiting illnesses (circle one)?

| **1** | **2** | **3** | **4** | **5** | **6** | **7** |
| --- | --- | --- | --- | --- | --- | --- |
| Infrequently |  |  | Occasionally |  |  | Regularly |

How consistently does your clinic provide **whole-person care** to patients with chronic, progressive, life-limiting illnesses?

| **1** | **2** | **3** | **4** | **5** | **6** | **7** |
| --- | --- | --- | --- | --- | --- | --- |
| Infrequently |  |  | Occasionally |  |  | Regularly |

How consistently does your clinic provide a **quality-of-life focus** to patients with chronic, progressive, life-limiting illnesses?

| **1** | **2** | **3** | **4** | **5** | **6** | **7** |
| --- | --- | --- | --- | --- | --- | --- |
| Infrequently |  |  | Occasionally |  |  | Regularly |

How consistently does your clinic provide **mortality acknowledgment** (including preparation for death, ACP and GOC discussions) to patients with chronic, progressive, life-limiting illnesses?

| **1** | **2** | **3** | **4** | **5** | **6** | **7** |
| --- | --- | --- | --- | --- | --- | --- |
| Infrequently |  |  | Occasionally |  |  | Regularly |

How consistently does your clinic use the **allied health team** (from your clinic) for patients with chronic, progressive, life-limiting illnesses?

| **1** | **2** | **3** | **4** | **5** | **6** | **7** |
| --- | --- | --- | --- | --- | --- | --- |
| Infrequently |  |  | Occasionally |  |  | Regularly |

How consistently does your clinic use **external allied clinician resources** (from the community) for patients with chronic, progressive, life-limiting illnesses?

| **1** | **2** | **3** | **4** | **5** | **6** | **7** |
| --- | --- | --- | --- | --- | --- | --- |
| Infrequently |  |  | Occasionally |  |  | Regularly |

How consistently does your clinic address the **needs of the caregiver** for patients with chronic, progressive, life-limiting illnesses?

| **1** | **2** | **3** | **4** | **5** | **6** | **7** |
| --- | --- | --- | --- | --- | --- | --- |
| Infrequently |  |  | Occasionally |  |  | Regularly |

How consistently does your clinic **assess and manage symptoms** of patients with chronic, progressive, life-limiting illnesses?

| **1** | **2** | **3** | **4** | **5** | **6** | **7** |
| --- | --- | --- | --- | --- | --- | --- |
| Infrequently |  |  | Occasionally |  |  | Regularly |
